# ‘The Bird Fights Its Way Out of the Egg’: A Phenomenological Study of Nurses’ Lived Experiences of Self-Care in South Korea’s Closed Psychiatric Wards

**DOI:** 10.3390/healthcare14030320

**Published:** 2026-01-27

**Authors:** Haejin Shin, Younjae Oh

**Affiliations:** 1 Department of Nursing, Youngnam Foreign Language College, 780-9 Namcheon-ro, Namcheon-myeon, Gyeongsan 38695, Gyeongsangbuk-do, Republic of Korea; 2 Research Institute of Nursing Science, College of Nursing, Hallym University, Hallymdaehakgil 1, Chuncheon 24252, Gangwon-do, Republic of Korea

**Keywords:** self-care, nurses, closed psychiatric wards, phenomenological study

## Abstract

**Background/Objectives**: Nurses working in closed psychiatric wards experience substantial psychosocial and spiritual burdens, emotional strain, and ethical tension due to continuous exposure to patients in crisis. As formal caregivers, nurses’ health and multidimensional well-being are essential for sustaining compassionate, dignity-preserving practice. However, the lived meaning of self-care within highly restrictive psychiatric environments remains insufficiently understood. This study explores how psychiatric nurses in South Korea experience and interpret self-care. **Methods**: A qualitative phenomenological design was used. Eight psychiatric nurses with more than three years of experience in closed psychiatric wards participated in in-depth, face-to-face interviews conducted between August 2018 and January 2019. Data were analysed using Colaizzi’s method to identify and synthesise essential themes. **Results**: Five categories captured the essence of nurses’ self-care experiences: (1) struggling to establish therapeutic roles as a psychiatric nurse; (2) conflating professional identity with ideals of good nursing; (3) recognising a gradual loss of motivation and hope to continue psychiatric nursing; (4) acknowledging the need to care for oneself and refocus on inner vitality; and (5) engaging in self-care through interactions with patients. Self-care was understood as a reflective, relational, and transformative process rather than as a set of stress-relief activities. **Conclusions**: Psychiatric nurses perceived self-care as an existential journey involving vulnerability, self-reflection, and renewal, which fostered both personal and professional growth. By framing self-care as an ethically grounded, relational practice that sustains therapeutic presence and safeguards moral and professional integrity, this study extends existing self-care literature beyond behavioural strategies.

## 1. Introduction

Nurses working in closed psychiatric hospitals care for individuals experiencing acute mental illness, aggression, and emotional instability. They spend long hours within locked wards, where the boundaries between therapeutic presence and personal vulnerability are continually tested [1,2]. Psychiatric nursing in such settings is inherently relational and ethically charged, requiring nurses to understand patients’ experiences from their perspectives while managing the strain of continuous exposure to psychological distress [3,4]. Although this context can foster deep human connection, it also contributes to emotional exhaustion, compassion fatigue, and professional ambiguity [5,6]. The ongoing challenge of maintaining humanity and professional integrity while delivering compassionate care has positioned self-care as a crucial yet underexplored issue in psychiatric nursing.

The concept of self-care among nurses has evolved from a personal coping strategy into a professional competency closely linked to ethical practice and quality of care [7,8]. The World Health Organization (WHO) defines self-care as an individual’s ability to promote health and manage health-related needs, with or without the support of a healthcare professional, thereby enabling an active role in maintaining overall well-being [9]. For nurses, self-care supports sustained compassion, emotional resilience, and responsiveness to patients despite prolonged exposure to suffering [10,11]. Within Watson’s Caritas framework [12] and care ethics [13], self-care is conceptualised as an ethically grounded practice that sustains therapeutic presence and dignity-preserving care. Consequently, nursing scholars emphasise that nurses’ self-care constitutes an ethical obligation, reflected in professional codes of ethics, and is essential for maintaining the capacity to deliver safe, high-quality care [8,14].

Although self-care in psychiatric settings extends beyond physical rest or emotional relief, research on psychiatric nurses’ self-care remains limited. Quantitative studies have largely focused on measured stress, burnout, or coping strategies, often overlooking the lived meaning of self-care as experienced by nurses themselves [15,16]. Empirical research in psychiatric and acute mental health nursing has primarily relied on survey designs to examine self-care related outcomes such as burnout, compassion fatigue, moral distress, and turnover intention [2,5,17], rather than the meanings and ethical negotiations that constitute self-care in everyday practice. Even where substantial psychosocial burden is documented in restrictive or locked settings, qualitative evidence explaining how nurses interpret and enact self-care within safety-focused routines and therapeutic relationships remains scarce.

Self-care is not a uniform behaviour but a lived, morally infused experience shaped by therapeutic relationships, ethical awareness, and a commitment to uphold the dignity of both nurses and patients [10,18]. The relative invisibility of nurses’ emotional and ethical needs points to a systemic imbalance in psychiatric care, where priorities may favour risk management, patient control, and symptom containment, leaving limited space for nurses’ existential well-being and moral integrity [1,2,5,19]. In South Korea, where closed psychiatric hospitals often face high patient loads, limited staffing, and persistent societal stigma toward mental illness, self-care is frequently overshadowed by institutional demands for efficiency and safety [20,21]. Despite these realities, little is known about how nurses in closed psychiatric wards understand and enact self-care as a relational and ethical practice amid ongoing tensions between safety and dignity and repeated exposure to distressing and aggressive situations. This gap constrains the development of context-sensitive organisational supports that address nurses’ emotional, ethical, and existential needs in restrictive psychiatric environments.

A phenomenological approach provides a rigorous framework for examining human experiences that are layered, ambiguous, and not readily accessible through quantitative inquiry [22,23,24]. Rather than treating self-care as a set of behavioural strategies, phenomenology allows exploration of how nurses make meaning of inner struggle, ethical tension, and renewal in everyday closed-ward practice. Guided by this methodological orientation, this study aimed to explore how self-care is lived, understood, and integrated into daily practice by nurses working in highly restrictive and emotionally demanding closed psychiatric wards, and to elucidate the essential structure of this experience.

## 2. Materials and Methods

### 2.1. Design

A qualitative phenomenological design was employed to explore how nurses working in closed psychiatric hospitals perceive and experience self-care in their daily practice. This design was selected to capture the depth and meaning of self-care as it is lived and interpreted by participants. Data were collected through individual, face-to-face interviews that invited reflective accounts of personal experience. Each interview opened with a broad, open-ended question: ‘Please describe your experiences of caring for yourself while working in a closed psychiatric ward’. Follow-up prompts, such as ‘Could you elaborate on that experience?’ and ‘What significance did it have for you?’, were used to encourage deeper reflection and clarification.

### 2.2. Participants

Participants were recruited using purposive sampling. The final sample comprised eight nurses from four closed psychiatric hospitals located in three provinces in South Korea. Based on recommendations from an expert panel that included psychiatric nurse practitioners, psychiatric nursing professors, and a phenomenologist, nurses with more than three years of clinical experience in closed psychiatric wards were eligible. This criterion was applied to ensure adequate exposure to the phenomenon under study. Data saturation was reached during the interview with the eighth participant, and data from all participants were included in the final analysis. Recruiting across multiple institutions and regions allowed for contextual variation in organisational and practice environments; however, institutional differences were not analysed as comparative categories.

Most participants were female nurses (75.0%). The average age was 36.6 years (range 30–47). Half of the participants had attained a graduate-level education (50.0%). Their average overall nursing experience was 11.0 years (range 6.2–15.0), and their experience working in closed psychiatric wards averaged 5.9 years (range 3.1–8.6) (Table 1).

### 2.3. Data Collection

Data were collected from August 2018 to January 2019. Data collection and preliminary analysis proceeded concurrently. After each interview, the research team reviewed the transcript and compared newly identified codes and meanings with those from prior interviews to assess whether substantively new themes or meaningful variations were emerging. During interviews, the interviewer observed and recorded non-verbal cues (e.g., facial expressions, tone, and gestures) and wrote reflective field notes capturing contextual observations and emerging analytic impressions. The interviews were conducted either in hospital counselling rooms or in participants’ homes, depending on individual preference, and were scheduled during off-duty hours or after work. Each participant took part in at least two interviews, each lasting approximately 60 to 90 min.

When participants found it challenging to articulate their experiences, the interviewer encouraged them to recall specific work situations and describe the emotions associated with caring for patients. The interviews were conducted in a conversational tone, and participants were invited to clarify or elaborate on ambiguous statements. Throughout the interviews, the interviewer maintained a neutral and empathetic stance, used minimal prompts, and allowed participants to speak freely. Audio recordings were transcribed verbatim and checked against the recordings to ensure accuracy. When additional clarification was required, brief follow-up telephone contacts were conducted, typically on two to three occasions, to confirm meanings and refine interpretations. Data collection ceased when saturation was judged to have occurred, that is, when additional interviews with the eighth participant yielded no substantively new themes or meaningful variations relevant to the phenomenon, as confirmed through team discussion.

### 2.4. Data Analysis

All interviews were transcribed verbatim by a professional transcriptionist and carefully checked for accuracy. This study adopted a descriptive phenomenological orientation aimed at elucidating the essential structure of nurses’ lived experiences of self-care in closed psychiatric wards. In keeping with descriptive phenomenology’s focus on the essence of experience, Colaizzi’s stepwise analytic method was used to derive meanings grounded in participants’ descriptions [25], supported by NVivo 10 qualitative analysis software. The analytic process involved several iterative steps: (1) reading and rereading all the transcripts to gain a comprehensive understanding of participants’ narratives; (2) identifying and extracting significant statements relevant to the phenomenon under study; (3) formulating meanings from these statements and confirming their validity through discussion within the research team; (4) clustering the meanings and integrating them into broader thematic categories; (5) constructing a detailed and exhaustive description of the emergent themes; and (6) deriving the fundamental structure that captured the essence of the lived experiences. Table 2 provides an illustrative example of the progression from significant statements to formulated meanings.

Preliminary analysis was undertaken in 2020, and the final analytic synthesis was completed in 2023 due to COVID-19-related disruptions that affected the availability of the author team. Transcripts were initially coded by the first author to identify significant statements and preliminary themes. The coding process and emerging thematic structure were reviewed by the co-authors, and discrepancies were discussed until consensus was reached. An audit trail, including coding notes and theme development memos, was maintained to enhance analytic transparency. The analytic process was conducted collaboratively among the research team to promote consistency and depth of interpretation.

### 2.5. Trustworthiness

The trustworthiness of this study was established in accordance with the evaluation criteria proposed by Lincoln and Guba [26]. Credibility was enhanced through prolonged engagement with participants, with at least two interviews conducted per participant, iterative interviewing with clarification through follow-up calls when needed, and team-based analytic discussions to ensure interpretations remained grounded in participants’ accounts. In addition, significant statements and their corresponding formulated meanings were reviewed by four participants through member checking to confirm that the analysis reflected their experiences. The researchers’ clinical experience in closed psychiatric wards, together with training in phenomenological philosophy and qualitative methods, supported sensitive interviewing and contextual understanding. To minimise the influence of pre-understandings, reflexive memoing and ongoing team discussions were used throughout data collection and analysis. Confirmability was supported through bracketing and reflexive documentation of assumptions and potential biases in field notes, alongside the maintenance of an audit trail that included coding notes, analytic memos, and records of theme development decisions. Consensus discussions among the research team were used to justify interpretive choices and ensure that findings were clearly traceable to the data. Dependability was supported by documenting the analytic procedure and decisions within the audit trail and through external examination, whereby five qualitative research experts reviewed the analytic process and findings. Based on their feedback, themes, theme clusters, and categories were refined to strengthen methodological coherence. Transferability was supported by providing a detailed description of the study context, participants, and data collection procedures, together with illustrative quotations that allow readers to evaluate the applicability of the findings to other settings.

### 2.6. Ethical Considerations

The study adhered to the ethical principles of the Declaration of Helsinki and received approval from the Institutional Review Board of Chosun University (IRB No. 2-1041055-AB-N-2018-36) on 24 July 2018. Participants were informed of the study’s purpose, procedures, confidentiality protections, and their right to withdraw at any time without repercussions. Written informed consent was obtained prior to participation, ensuring voluntary and ethically appropriate involvement.

## 3. Results

Data analysis yielded five categories, comprising 14 theme clusters and 27 themes illustrating the multidimensional nature of psychiatric nurses’ self-care experiences (Table 3).

### 3.1. Struggling to Find Therapeutic Roles of a Psychiatric Nurses

In the early stages of working in closed wards, nurses experienced fear and uncertainty in an unfamiliar and highly controlled environment. Over time, this fear shifted toward compassion, advocacy, and more intentional therapeutic engagement, as nurses found meaning through relationships that supported patients’ recovery.

#### 3.1.1. Fear Originating from a Closed Space

Nurses described the closed ward as an alien and confining environment. Locked doors, restricted movement, and constant surveillance generated discomfort comparable to being inside a detention facility. Many participants expressed initial fear that patients might behave unpredictably or violently, shaped in part by media portrayals and stereotypes about psychiatric hospitals. This fear led to emotional distance and resulted in cautious, guarded interactions during the early stages of care.

#### 3.1.2. Compassion Toward Psychiatric Patients

As nurses became more familiar with patients’ conditions and daily struggles, fear gradually transformed into compassion. Witnessing patients’ loss of freedom and autonomy evoked feelings of pity and empathy. Nurses expressed a desire to stabilise their patients, support their recovery, and, where possible, prevent repeated hospitalisations. One nurse reflected:

*It’s such a suffocating space. It’s basically a prison. When patients say it feels like a prison, I can’t deny it. (…) There’s really nothing for them to do in here compared with the outside. (…) I try to offer a few words of empathy, but I can’t keep doing that endlessly. So I tell them, ‘Let’s try to find something—there’s this, and there’s that’. Even so, if I were admitted here, like they are, I think I would feel like I couldn’t breathe.*

*(Participant 7)*

#### 3.1.3. Discovering the Role of Patient Advocate

Through daily encounters, nurses came to realise that their role extended beyond symptom management alone. They recognised patients’ vulnerability within a restrictive system and felt compelled to stand on their side. Nurses described themselves as advocates who protected patients’ dignity, articulated patients’ concerns, and sought fairness in treatment related decisions. Advocacy emerged as a meaningful component of their therapeutic identity, as evident from this excerpt:

*There are so many things that patients with mental illness here cannot do, even if they want to. This is a closed ward, after all, and there are many rules—always justified as being ‘for the patients’. (…) I believe I should be the one who helps make that possible. I’m not a doctor; I’m a nurse. So I should identify and do everything I can for the sake of my patients.*

*(Participant 6)*

#### 3.1.4. Striving to Engage in Therapeutic Interaction with Patients

As nurses grew in confidence, they began to engage more intentionally in therapeutic interactions with patients. They became aware that their presence, tone, and behaviour could have healing effects. Moments in which patients responded positively were experienced as rewarding and reaffirmed the meaning of their work. These experiences strengthened nurses’ belief that psychiatric nursing is fundamentally relational. One nurse described:

*There was a patient who persistently refused meals due to psychotic symptoms. By chance, I kept caring for them during my shifts, so I began to talk with them and try a more therapeutic approach. As our relationship developed, we made concrete plans—how much they would try to eat each day—and we checked in, encouraged each other, and used small rewards. That experience became a turning point for me. I thought, ‘This is what it means to be a psychiatric nurse. This work is built on relationship.’*

*(Participant 4)*

### 3.2. Confusing Professional Identity with Good Nursing

Nurses described confusion about what constituted ‘good nursing’ as they navigated uncontrolled psychiatric symptoms, unexpected aggression, and ethical tensions between autonomy and safety within rigid ward routines. These challenges often blurred their professional identity and intensified moral distress.

#### 3.2.1. Distress Related to Difficulties in Managing Psychiatric Symptoms

Nurses frequently encountered patient symptoms that were unpredictable, intense, or resistant to treatment. They described feeling overwhelmed by the emotional labour and disappointed when they were unable to provide recovery oriented care due to heavy workloads or systemic constraints. This discrepancy between idealised nursing practice and clinical realities generated feelings of guilt and inadequacy. This distress is illustrated in the following excerpt:

*The patient seems indifferent to their own symptoms, with no energy or will to live. Seeing them like that, I often find myself asking, ‘What am I even doing?’ Even when I poured 80 out of my 100 units of energy into this person, it made no difference. Realising that, I felt such a sense of futility as a therapeutic nurse. It made me wonder whether I had reached my own limits.*

*(Participant 5)*

#### 3.2.2. Uncertainty About How to Respond to Patients’ Unexpected Aggressive Behaviours

Nurses described moments in which patients suddenly became aggressive or violent. In these moments, they felt unprepared and uncertain about how to respond in a therapeutic manner. Some reported feeling more like guards or controllers than therapeutic professionals. These experiences undermined their sense of professional competence and further blurred their professional identity. One nurse explained:

*We’re taught to interpret violence as part of the patient’s symptoms, but after being assaulted repeatedly, that’s not easy to do. Even noticing the early warning signs makes me step back—not because I dislike the patient, but because fear comes before my professional response. Once, I was hit so hard that my face swelled and I struggled to eat for days. Experiences like that shake my sense of competence and make me question who I am at work—as a nurse, but also as a person.*

*(Participant 1)*

#### 3.2.3. Struggling to Balance Incompatible Ethical Values

Nurses frequently encountered ethical tensions, especially between respecting patient autonomy and ensuring safety. The rigid routines and highly controlled environment of closed wards at times conflicted with nurses’ therapeutic intentions. Nurses described feeling caught between institutional demands and their desire to provide dignified, patient-centred care. This internal struggle contributed to moral distress and confusion about their professional role, as is clear from this excerpt:

*Because of that, the ward prioritises safety and protection over allowing patients to make their own decisions about their treatment or health. In those moments, I often find myself questioning whether this is truly the right approach as a nurse… it’s agonising, but it’s difficult to speak up against the head nurse or the organisation. The atmosphere makes it hard, and, to be honest, I lack the courage.*

*(Participant 2)*

### 3.3. Recognising Oneself Losing the Drive and Hope to Continue as a Psychiatric Nurse

Over time, nurses experienced emotional and existential exhaustion that led to withdrawal, emotional blunting, and despair. This gradual erosion of vitality destabilised their sense of professional growth and undermined confidence in their ability to continue working as psychiatric nurses.

#### 3.3.1. Fear of Seeing Oneself Withdraw from Patients

Nurses became aware that their energy was diminishing to the point where engagement with patients was limited to task focused interactions. They described feeling hollow and emotionally detached and, at times, deliberately avoiding patients to conserve their remaining energy. This withdrawal was experienced as frightening, as it signalled a departure from their core beliefs about what constitutes good nursing.

#### 3.3.2. Distress over Becoming Desensitised to the Human Dignity of Psychiatric Patients

With repeated exposure to patients whose conditions showed little improvement, nurses reported emotional numbness. Some described moments when patients seemed like ‘lifeless bodies,’ making it difficult to recognise their humanity. Nurses experienced distress, guilt, and sorrow regarding their own emotional blunting and the ambivalence they experienced toward patients. The following excerpt illustrates situations that generated emotional distress for nurses:

*The scariest part is this: when I was a new nurse, I used to resent a senior nurse who seemed indifferent to patients’ suffering, showing no compassion or respect for them. I even thought to myself that she wasn’t a real nurse. But now, in my seventh year, I saw a patient who kept breaking the ward rules and ended up being physically restrained. When I looked at him suffering in restraints, I felt nothing. I realised that I had become exactly like that senior nurse I once criticised. I feel ashamed of myself, and I don’t like who I’ve become.*

*(Participant 4)*

#### 3.3.3. Collapse of Professional Self-Esteem and Sinking into Despair

As emotional exhaustion persisted, nurses’ confidence gradually eroded. Aspirations for professional development diminished and were replaced by feelings of failure, stagnation, and self-doubt about their ability to continue working as psychiatric nurses. Nurses described sinking deeper into despair and feeling unable to release accumulated negative emotions, which at times spilled over into interactions with others. One nurse explained how repeated exposure to patients’ distressing narratives accumulated emotionally over time:

*Patients tell nurses very heavy stories—about suicide, self-harm, and sometimes horrific thoughts of harming others, whether delusional or real. When you listen to this day after day, it piles up, layer upon layer, and I feel emotionally worn down. Then I start wondering, ‘How long can I endure this?’ and ‘Am I even the right person to be a psychiatric nurse?’ Some mornings on the way to work, I feel like an animal being dragged to a slaughterhouse. It feels unbearable to go in and hear those painful stories again, and I doubt whether someone like me can be of any therapeutic help. Over time, it feels as if I’m sinking into a swamp I can’t escape.*

*(Participant 3)*

### 3.4. Realising the Need to Care for and Refocus on One’s Inner Vitality

As exhaustion deepened, nurses increasingly came to view self-care as essential for restoring inner vitality and sustaining their capacity to care for patients.

#### 3.4.1. Reconnecting with One’s Inner Vitality

Experiences of emotional exhaustion prompted nurses to turn inward and actively seek peace, calmness, and emotional recovery. They described engaging in personal activities to replenish depleted energy and release accumulated emotions. Through this inward reflection, nurses recognised that their inner selves remained intact, which enabled them to reconnect with a sense of vitality.

*I realised that nothing would change if I just kept stressing and suffering because of this job. I thought, ‘I need to do something I actually enjoy’. It was like feeling the need to rediscover myself again… to confirm that I still existed. (…) When I run marathons, I sometimes become extremely out of breath. In those moments, I think, ‘Ah, I’m alive’. Focusing on myself like that made me feel a little better.*

*(Participant 5)*

#### 3.4.2. Awakening Awareness of the Importance of Self-Care

Nurses came to recognise that self-care was not optional but essential, and that replenishing their own emotional resources was necessary in order to provide meaningful care to patients. This realisation marked a shift from self-neglect to intentional and reflective self-care, reinforcing the reciprocal relationship between caring for oneself and caring for others, as exemplified by the following excerpt:

*If I’ve chosen to work as a psychiatric nurse in a closed ward and plan to continue on this path, I need fuel—energy. If I compare it to a car, it’s like gasoline. Focusing on myself and taking care of myself… (pointing at himself)—that gasoline is my motivation. (…) And no one else fills up my tank for me. I have to do it myself. (…) And my car has to run properly so it doesn’t cause problems for the other cars on the road.*

*(Participant 8)*

### 3.5. Caring for Oneself Through Interactions with Patients

Meaningful nurse–patient encounters became a restorative source of motivation and self-growth, helping nurses reaffirm their therapeutic identity and capacity to continue in practice.

#### 3.5.1. Professional Growth as a Psychiatric Nurse

Meaningful encounters with patients reignited nurses’ inner motivation and reminded them of their original reasons for entering psychiatric nursing. By rediscovering the inner drive that supported sustained therapeutic practice, nurses reaffirmed their sense of responsibility and commitment to becoming better psychiatric nurses. These experiences helped them re-establish professional confidence, as is clear from this excerpt:

*How could I genuinely care for my patients if I myself am shaken? I thought, ‘If I’m anxious, how can I care for someone who is anxious?’ I realised that I need to clearly identify what helps me feel at ease. I came to understand that taking care of myself is, in a way, my responsibility as a nurse and as a professional caregiver.*

*(Participant 1)*

#### 3.5.2. Experiencing Self-Growth Through Interactions with Patients

Patients served as mirrors for nurses’ own growth. Nurses observed themselves striving once again to be compassionate and competent caregivers, reflecting both personal and professional development. Through reciprocal interactions with patients, they gained insight into themselves, recognised internal changes, and discovered that self-growth could emerge from the therapeutic relationship itself. Nurses described self-care as closely intertwined with caring for patients: ‘The more I understood my patients, the more I understood myself’. This reflective process helped them rediscover their professional identity and vitality, symbolised by the metaphor *‘The bird fights its way out of the egg’.* The following excerpt clarifies this insight:

*When some patients told me that the conversations during which I listened to their stories and comforted them gave them hope in life, I felt a deep sense of fulfilment. That was when I realised, ‘Ah, I’m not the only one helping. The patients are helping me too’. (…) Among the patients, there are those who, despite repeated admissions and discharges, still try hard to get better. When I see them—and their families or children—doing their best even in such difficult circumstances, I find myself wondering, ‘Why am I like this when they are trying so hard?’ It made me realise that I also need to overcome things on my own.*

*(Participant 3)*

### 3.6. Essential Structure of Nurses’ Lived Experiences of Self-Care in South Korea’s Closed Psychiatric Wards

Self-care for nurses working in closed psychiatric wards emerged as a deeply relational and existential process shaped by fear, emotional depletion, ethical tension, and the gradual rediscovery of inner vitality. When first entering closed wards, nurses confronted a restrictive and unfamiliar environment. Locked doors, rigid routines, and unpredictable patient behaviours evoked fear, heightened vigilance, and emotional distance. Over time, as nurses witnessed patients’ suffering and loss of freedom, fear gradually transformed into compassion. Nurses begin to recognise their role as advocates responsible for protecting patients’ dignity and rights within a confined system. Therapeutic encounters with patients provided moments of connection that affirmed the meaningfulness of nurses’ presence.

However, these therapeutic intentions often collide with the realities of psychiatric care. Nurses struggle to manage uncontrolled symptoms, respond to sudden aggression, and navigate conflicts between patient autonomy and safety. Such dilemmas evoke guilt, inadequacy, and doubt about whether they are practising what they perceive as good nursing. Over time, constant exposure to chronic illness, emotional labour, and institutional constraints erodes nurses’ professional identity. Nurses described withdrawing from patients, becoming desensitised to human dignity, and questioning the kind of nurse and person they had become. Feelings of stagnation, shame, and helplessness accumulated, leaving them emotionally exhausted and unable to release pent-up distress.

This deterioration, together with nurses’ recognition of their own vulnerability, led to a crucial realisation that sustaining care for others first required reclaiming oneself. Nurses turned inward in search of peace and emotional restoration, attempting to reconnect with their inner vitality and recognising that no one else could replenish their emotional energy. Self-care thus emerged not as a luxury but as an ethical and professional responsibility, forming an essential foundation for therapeutic presence and effective caregiving.

Ultimately, nurses discovered that self-care and patient care are intertwined. Interactions with patients, including listening to their stories, witnessing their efforts to recover, and sensing their appreciation, became sources of renewed meaning and motivation. Patients act as mirrors, reflecting nurses’ personal and professional growth. These reciprocal relationships enabled nurses to rediscover their inner drive and reaffirm their commitment to compassionate psychiatric nursing. Through such encounters, they regained a sense of hope, purpose, and identity, symbolised by the metaphor ‘The bird fights its way out of the egg’.

Throughout this journey, self-care was understood not as a discrete set of actions but as a dynamic and cyclical process of losing and reclaiming oneself. It involved confronting fear, confusion, and despair, reconnecting with inner vitality, and recognising the reciprocity inherent in therapeutic relationships. Through this evolving process, nurses working in closed psychiatric wards rebuilt both their humanity and their capacity to care with authenticity and resilience.

## 4. Discussion

This phenomenological study explored how nurses working in closed psychiatric wards in South Korea experience self-care within an environment characterised by structural confinement, emotional intensity, and ethically complex therapeutic relationships. The findings reconceptualise self-care beyond an individual coping strategy by demonstrating it as a relational and ethically grounded practice through which nurses sustain therapeutic presence and protect moral and professional integrity under restrictive conditions. The essential structure of this experience emerged as a trajectory from fear and confusion to emotional collapse, followed by self-reflection and, ultimately, personal and professional growth through mutual and responsive caring relationships.

Within the broader caregiver continuum, psychiatric nurses can be conceptualised as formal caregivers who provide professional caregiving within institutional healthcare settings, distinct from informal caregiving by family members. This framing underscores that, as formal caregivers, nurses’ health and multidimensional well-being in closed wards are shaped not only by individual coping efforts but also by organisational conditions that influence opportunities for ethically responsive care. Viewed through a caregiver health lens, this trajectory illustrates how sustained psychosocial strain and existential concerns in locked settings can erode multidimensional well-being, and how relationally grounded self-care can support resilience among nurses. Although the interviews were conducted between 2018 and 2019, the core structural and ethical conditions of closed psychiatric wards, including restrictive routines, risk management demands, and sustained emotional labour, remain salient. Recent literature continues to document similar burdens and ethical tensions in closed mental health settings, supporting the ongoing relevance of the present findings [17,27].

Nurses initially confronted closed ward environments with fear, uncertainty, and emotional distance, responses commonly reported in studies of psychiatric nursing in restrictive settings where unpredictable behaviour and locked wards intensify vigilance and anxiety [2,28]. Studies conducted during the COVID-19 period in closed psychiatric wards have similarly documented heightened anxiety, fear of infection, and feelings of entrapment, suggesting that locked settings can amplify psychological burden and threaten nurses’ sense of safety and control [18,29]. As nurses in this study encountered patient suffering and loss of autonomy, fear gradually transformed into compassion and a growing awareness of the need for patient advocacy. This pattern aligns with research emphasising the relational and moral dimensions of psychiatric nursing, in which advocacy emerges from understanding patients’ lived experiences rather than solely from procedural duties [30,31].

The shift from fear to compassion illustrates that therapeutic nursing identity develops through ongoing emotional negotiation as nurses recognise the humanity and vulnerability of their patients. This transformation can be understood in ethical terms. Levinas argues that encountering the suffering face of the Other calls forth a compassion that is both moral and deeply human [32]. The progression observed among nurses in this study resonates with scholarship in psychiatric and relational ethics, which highlights recognition of vulnerability as foundational to the development of empathic therapeutic presence [28,33]. In this sense, movement toward compassion represents not only an emotional transition but also the emergence of a morally grounded therapeutic identity that may function as a source of resilience.

Nurses struggled with conflicting expectations of what it means to practice ‘good nursing’ in closed wards. Managing uncontrolled symptoms, responding to sudden aggression, and adhering to rigid institutional routines created tension between safety-oriented and recovery-oriented values. Similar dilemmas have been reported in studies of ethical decision making in psychiatric settings, where nurses navigate competing imperatives of autonomy, safety, and therapeutic engagement [3,34]. This confusion reflects the inherently dual nature of psychiatric nursing, situated between custodial control and therapeutic care [20]. Moral distress also arises when nurses encounter institutional constraints, such as rigid ward routines, that impede their efforts to provide good nursing. This observation echoes earlier research demonstrating that such environments can undermine nurses’ ethical agency [3,5,34]. Moreover, this issue remains unresolved. A recent systematic review on moral distress among acute mental health nurses reported that moral distress is highly prevalent in these settings and is associated with poorer outcomes for nurses, patients, and organisations, while evidence for effective interventions remains limited [17]. Together, these findings suggest that tensions between safety and dignity are organisationally produced, and that self-care is experienced as a means of maintaining an ethical stance amid competing institutional demands. Overall, this phenomenon indicates that professional ambiguity is not an individual failing but a structural consequence of practising within restrictive systems.

Repeated exposure to patient suffering, clinical stagnation, and institutional rigidity led to emotional withdrawal, numbness, and despair among nurses. Their descriptions of losing sensitivity to dignity and feeling stagnant mirror empirical findings on burnout, compassion fatigue, and desensitisation among psychiatric nurses [5,35]. However, the present findings extend these constructs by revealing an existential and moral dimension. Nurses described not only emotional depletion but also a threatened moral self-understanding when withdrawal from patients conflicted with their commitment to dignity-preserving care. They reported psychological distress, including guilt and a troubled conscience, arising from the awareness that withdrawing from patient care violated the dignity of those for whom they felt responsible. This interpretation is consistent with a recent systematic review of ethical issues perceived by nurses, which identified failure to provide dignified care not only as a central value of nursing but also a fundamental normative standard of practice [18].

A turning point emerged when nurses recognised the need to focus on their own well-being, as they experienced emotional depletion and perceived threats to their professional identity, realising that their vulnerability carried ethical implications for their practice. This shift reflects theoretical perspectives that frame self-care as an ethical obligation rather than as a coping behaviour alone [12,13]. Nurses described intentionally refocusing on inner vitality and acknowledging the importance of maintaining emotional equilibrium to safeguard both their own integrity and patient safety. This interpretation aligns with the literature emphasising that self-care supports emotional regulation, prevents moral erosion, and preserves the capacity for compassion [8,36]. Nurses’ metaphor of ‘refuelling’ resonates with caring science, particularly Watson’s theory of human caring, which underscores the restoration of mind–body–spirit as a prerequisite for authentic caring [12,36]. This suggests that self-care may function as a form of moral restoration whereby restoring inner resources enables nurses to remain ethically responsive and therapeutically engaged within restrictive environments.

Finally, nurses came to realise that interactions with patients nurtured their own well-being, highlighting the reciprocal and restorative dimensions of the nurse–patient relationship. Meaningful encounters, such as witnessing patients’ efforts to recover or hearing expressions of gratitude, renewed nurses’ sense of purpose and strengthened their professional identity. This finding aligns with nursing theories that conceptualise caring as a mutual process in which dignity emerges through relational responsiveness within caregiving encounters. From this perspective, self-care becomes an ethical responsibility, as caring for oneself sustains the capacity to care ethically for others [31,37]. Gastmans [38], drawing on Tronto’s concept of care receiving, emphasises that although reciprocity is a crucial moral dimension of nursing care, the provision of care should not depend on the patient’s cognitive or emotional capacity to respond. Nurses are called to provide care grounded in respect for human dignity, regardless of the patient’s status or illness. This perspective clarifies why nurses in this study experienced relational encounters as ethically meaningful moments that contributed to restoring their own well-being.

Interactions with patients also served as sources of self-growth, enabling nurses to strive once again to become the practitioners they aspired to be. Such mutual affirmation supports research demonstrating that therapeutic relationships can mitigate burnout and reinforce professional commitment [11,33]. In this sense, self-care is not separate from patient care; rather, meaningful relational engagement constitutes a central pathway through which nurses restore themselves emotionally and professionally.

## 5. Implications and Limitations

### 5.1. Implications

The findings of this study have important implications for psychiatric nursing and for supporting nurses as formal caregivers in restrictive mental health settings. They highlight the psychosocial and spiritual burden experienced by nurses in closed wards and demonstrate that self-care is closely linked to caregiver health, multidimensional well-being, and resilience. Therefore, nurses’ emotional and existential vulnerability should be recognised as an inherent feature of work in restrictive psychiatric wards rather than as an individual weakness.

Because nurses perceive self-care as an ethical and professional responsibility, educational and organisational systems should promote and support self-care not merely as stress management but as a prerequisite for sustaining ethical and therapeutic nursing practice. Nurses’ early uncertainty regarding their therapeutic roles also highlights the need to reinforce relational presence, therapeutic communication, advocacy, and dignity-preserving care as essential components of psychiatric practice, even within safety-focused routines.

Organisational responsibility for protecting and promoting nurses’ professional and moral integrity should be explicitly operationalised through embedded support systems. For example, organisations can provide structured post-incident debriefings, protected time for reflective dialogue, timely ethics support such as consultation, ethics rounds, or case-based debriefings, and confidential emotional support pathways such as counselling or an Employee Assistance Program (EAP). These interventions may help prevent emotional withdrawal and erosion of professional identity, while strengthening nurses’ well-being and resilience.

Although grounded in the South Korean context, these implications are likely transferable to other healthcare systems that rely on locked or restrictive psychiatric care during acute crises, where nurses similarly face safety–dignity tensions, exposure to aggression, and sustained emotional labour. Overall, the staged trajectory identified in this study may inform preventive and supportive caregiver-support interventions tailored to nurses’ evolving needs, guiding when and how support is offered across professional development in closed wards, from early-stage training and mentorship to restorative reflective practices.

### 5.2. Limitations

This study has several limitations that should be considered when interpreting the findings. First, the interviews were conducted between 2018 and 2019, which may limit contemporaneity. Nevertheless, the recent literature indicates that similar burdens and ethical tensions continue to characterise closed mental health settings. Second, the research was conducted in closed psychiatric wards in South Korea, a context marked by high levels of structural restriction and culturally specific perceptions of mental illness. This may limit the transferability of the findings to other psychiatric environments or healthcare systems. Third, as is typical of phenomenological research, the sample size was small and purposively selected to achieve depth rather than breadth. Although this approach generated rich data, the experiences described may not reflect those of all psychiatric nurses, particularly those working in community or outpatient settings. Finally, the study relied on self-reported narratives obtained through in-depth interviews. Participants’ accounts may have been influenced by recall bias, emotional state, or reluctance to disclose sensitive experiences. Additionally, despite efforts to bracket preconceptions, complete elimination of researchers’ assumptions is unattainable, and interpretation may have been shaped by the research team’s backgrounds in psychiatric nursing and ethics.

## 6. Conclusions

This study revealed that psychiatric nurses working in closed wards experience self-care as an evolving process shaped by vulnerability, ethical tension, emotional collapse, and eventual renewal through reflection and reciprocal caring relationships. Within restrictive closed ward contexts, self-care was experienced as a relational and dignity-oriented practice that sustains therapeutic presence and moral integrity while enabling continued engagement in care. Nurses’ well-being was closely intertwined with organisational structures, moral challenges, and the restorative potential of mutual nurse–patient encounters. Although the data were collected between 2018 and 2019, the core structural and ethical conditions of closed psychiatric wards remain salient, supporting the ongoing relevance of these findings. Supporting nurses’ emotional, ethical, and existential needs is therefore essential to foster resilient, compassionate, and dignity-enhancing psychiatric care. To inform practice and education, healthcare organisations should embed preventive and supportive systems for nurses working in closed wards and evaluate their effects on nurses’ well-being, retention, and quality of care in restrictive psychiatric settings.

## Figures and Tables

**Table 1:** General characteristics of the participants.

No	Gender	Age (Years)	Marital Status	Educational Level Attained	Nursing Experience (Years)	Work Experience in a Closed Psychiatric Ward
1	Female	39	Unmarried	Graduate degree	13.6	7.6
2	Female	34	Unmarried	Bachelor’s degree	11.4	5.4
3	Female	42	Unmarried	Bachelor’s degree	13.0	3.1
4	Female	34	Unmarried	Graduate degree	10.6	8.6
5	Male	30	Unmarried	Bachelor’s degree	7.4	5.4
6	Female	36	Married	Graduate degree	15.0	4.0
7	Female	47	Married	Graduate degree	11.0	8.0
8	Male	31	Unmarried	Bachelor’s degree	6.2	5.4

**Table 2:** Illustrative example of Colaizzi’s analytic process (significant statements to formulated meanings).

Significant Statement (Excerpt)	Formulated Meaning	Theme Cluster	Category
*“When I looked at him suffering in restraints, I felt nothing. I realised that I had become exactly like that senior nurse I once criticised.” (Participant 4)*	Recognising emotional numbness and moral discomfort when observing patient suffering, accompanied by fear of becoming indifferent and losing one’s caring identity.	Distress related to becoming desensitised to the human dignity of psychiatric patients	Recognising oneself losing the drive and hope to continue as a psychiatric nurse
*“If I’m anxious, how can I care for someone who is anxious? … taking care of myself is, in a way, my responsibility as a nurse.” (Participant 1)*	Understanding self-care as a professional and ethical responsibility that enables therapeutic presence and emotional regulation.	Awakening awareness of the importance of self-care	Realising the need to care for and refocus on one’s inner vitality

Excerpts are verbatim participant quotations and are italicized.

**Table 3:** Summary of themes, theme clusters, and categories.

Categories	Theme Clusters	Themes
Struggling to find the therapeutic role of a psychiatric nurse	Fear originating from a closed space	Feeling discomfort in an environment perceived as a confinement facility Fear of patients’ unpredictable behaviour
	Compassion toward psychiatric patients	Feeling pity and empathy for patients deprived of freedom Desire to help patients recover and not return to the hospital
	Discovering the role of patient advocate	Recognising the need to stand on the patient’s side within the therapeutic team Wanting to protect patients’ rights and dignity in restrictive settings
	Striving to engage in therapeutic interaction with patients	Realising that one’s presence has healing value Experiencing fulfilment through effective therapeutic relationships
Confusing professional identity with good nursing	Distress related to difficulties in managing psychiatric symptoms	Feeling overwhelmed when facing complex or unpredictable psychiatric symptoms Feeling guilty about being unable to provide recovery-oriented care
	Uncertainty about how to respond to patients’ unexpected aggressive behaviours	Feeling unsure about how to act when patients suddenly become aggressive Feeling like a guard rather than a therapeutic professional
	Struggling to balance incompatible ethical values	Conflicts between patient autonomy and safety Feeling trapped by the ward’s rigid routines
Recognising oneself losing the drive and hope to continue as a psychiatric nurse	Fear of seeing oneself withdraw from patients	Depletion of emotional energy and withdrawal from patients
	Distress over becoming desensitised to the human dignity of psychiatric patients	Difficulty perceiving patients’ dignity Feeling distressed over emotional numbness Experiencing mixed emotions of guilt, irritation, and sorrow
	Collapse of professional self-esteem and sinking into despair	Losing confidence and hope for professional growth Feeling trapped in persistent despair with no perceived escape Emotional exhaustion affecting interactions with others
Realising the need to care for and refocus on one’s inner vitality	Reconnecting with one’s inner vitality	Seeking inner peace and replenishing depleted emotional energy Confirming that one’s inner self remains alive
	Awakening awareness of the importance of self-care	Realising the need for self-care in order to care for patients
Caring for oneself through interactions with patients	Professional growth as a psychiatric nurse	Rediscovering the inner drive that sustains steady therapeutic practice Recognising renewed responsibility and commitment to becoming a better psychiatric nurse
	Experiencing self-growth through interactions with patients	Realising personal growth by recognising renewed efforts to become a good nurse for patients

## Data Availability

The data generated and analysed during this study are not readily available because ethical approval for use relates only to the research team. Requests to access the datasets should be directed to the corresponding author upon reasonable request, subject to ethical approval.
